# Molecular Modeling of Metabolism for Allergen-Free Low Linoleic Acid Peanuts

**DOI:** 10.1007/s12010-012-9821-6

**Published:** 2012-08-24

**Authors:** Godson O. Osuji, Tassine K. Brown, Sanique M. South, Dwiesha Johnson, Shanique Hyllam

**Affiliations:** CARC, Prairie View A&M University, P.O. Box 519-2008, Prairie View, TX 77446 USA

**Keywords:** Glutamate dehydrogenase-synthesized RNA, Partial downregulation, HPLC, Northern hybridization, mRNA permutation

## Abstract

It is necessary to eliminate linoleic acid and allergenic arachins from peanuts for good health reasons. Virginia-type peanuts, harvested from plots treated with mineral salts combinations that mimic the subunit compositions of glutamate dehydrogenase (GDH) were analyzed for fatty acid and arachin compositions by HPLC and polyacrylamide gel electrophoresis, respectively. Fatty acid desaturase and arachin encoding mRNAs were analyzed by Northern hybridization using the homologous RNAs synthesized by peanut GDH as probes. There were 70–80 % sequence similarities between the GDH-synthesized RNAs and the mRNAs encoding arachins, fatty acid desaturases, glutamate synthase, and nitrate reductase, which similarities induced permutation of the metabolic pathways at the mRNA level. Modeling of mRNAs showed there were 210, 3,150, 1,260, 2,520, and 4,200 metabolic permutations in the control, NPKS-, NS-, Pi-, NH_4_Cl-, and PK-treated peanuts, respectively. The mRNA cross-talks decreased the arachin to almost zero percent in the NPKS- and PK-treated peanuts, and linoleate to ∼18 % in the PK-treated peanut. The mRNA cross-talks may account for the vastly reported environmentally induced variability in the linoleate contents of peanut genotypes. These results have quantitatively unified molecular biology and metabolic pathways into one simple biotechnology for optimizing peanut quality and may encourage small-scale industry to produce arachin-free low linoleate peanuts.

## Introduction

Peanut contributes more than four billion dollars annually to the US economy. But arachins [1, 2] and some unsaturated fatty acids [3] are undesirable components of peanut because they pose health risks to many peanut consumers. Arachins are the allergenic proteins of peanuts. They induce crippling whole-body anaphylactic reactions in sensitive patients [4]. Worldwide, up to 0.6 % of humanity is sensitive to allergic arachins [5]. Although it is now possible to eliminate arachins from peanut by genetic engineering procedures [1], the preponderant peanuts in grocery stores and restaurants are laden with arachins. Another health concern of peanut is its high content of linoleic acid that increases the synthesis of low density lipoprotein cholesterol thereby increasing the risk of heart attacks [6]. Although it is now possible through genetic engineering and plant breeding procedures to silence fatty acid desaturases that catalyze the introduction of double bonds into the hydrocarbon chain [7], the preponderant peanuts in the grocery store possess high linoleate [8]. The idea is to develop a general purpose biotechnology for producing allergen-free low linoleate peanut.

Although peanut protein compositions are affected by the maturity stages of the peanut [9], and unsaturated fatty acid compositions are affected by epistatic, pleiotropic, environment × genotype interactions under which the peanuts were produced [10–12], the molecular basis of the responses of arachins and fatty acid desaturases to environmental conditions have not been investigated. The variations of the oleate/linoleate (O/L) ratios per peanut genotype [13–16] have not been explained in terms of the molecular biology of fatty acid desaturases. Also, although the genetics of arachins [17] and fatty acid desaturases [11, 18] and chemistry of desaturation reaction [19] have been studied in detail, their molecular relationships to other metabolic pathways (CO_2_ and nitrogen assimilation, glycolysis, saccharide, and nucleotide biosyntheses etc.) have not been uncovered. Understanding the molecular relationships between arachin and unsaturated fatty acid accumulation may facilitate the development of simple models and biotechnologies for eliminating linoleic acid and arachins from normal peanut.

Many peanut allergens have been identified, and three of them (Ara h1, Ara h2, and Ara h3) have been characterized at the protein, cDNA, and genomic DNA levels [1]. Ara h1 is the major allergenic protein and it exists as isoforms of ∼65 kDa, with encoding mRNAs 1,900–2,300 bp [17]. Ara h2 (∼18 kDa) and Ara h3 (∼60 kDa) are smaller in molecular size and in percent of peanut protein composition than Ara h1 although Ara h2 is a more potent allergen than Ara h1 and Ara h3 [1]. Linoleic acid phenotypes in peanut [15] are controlled by recessive fatty acid desaturase genes (FAD2A and FAD2B). In terms of the O/L ratios, Virginia and runner-type cultivars have been classified into four genotypes: OL_1_OL_1_OL_2_OL_2_ for the wild type, ol_1_ol_1_OL_2_OL_2_ for homozygous A genome, OL_1_OL_1_ol_2_ol_2_ for the homozygous B genome, and ol_1_ol_1_ol_2_ol_2_ for the double mutation in both A and B genomes [16]. The mRNAs encoding the fatty acid desaturases range from 1,400 to 3,500 kb suggestive of possible multiple copies of the genes [8].

Permutation of mRNA by glutamate dehydrogenase (GDH)-synthesized RNA [20] opens a new gateway for investigating the possible molecular signaling between natural environment (mineral nutrients, etc.), mRNAs encoding arachins and fatty acid desaturases (peanut genetics), and peanut metabolism (biological system). Peanut GDH is very active in the synthesis of RNA [21, 22]. Earlier studies on GDH kinetics had observed the signal integration/discrimination [23] and biomass enhancement [24] functions of the enzyme. Molecular studies have demonstrated that the signal integration/discrimination function has been occurring since evolutionary time [25]. This emphasizes the importance of GDH-synthesized RNA in the modification of gene expression and metabolism. The principles and practice of the induction of GDH-synthesized RNAs have been applied to explain some hitherto inexplicable biological phenomena including metabolic detoxification of xenobiotics in plants [26]; the regulation of fatty acid contents by mRNA encoding lipoxygenase [27]; the regulation of cellulosic biomass and fatty acid accumulation by mRNAs encoding acetyl CoA carboxylase, nitrate reductase, phosphate translocator [28]; and doubling of peanut yield through permutation of mRNAs encoding the enzymes of primary metabolism [20]. In all cases, there were agreement between the permuted mRNAs and the yields of peanut fatty acids and cellulosic biomass. GDH-synthesized RNAs are ideal for modeling arachin and fatty acid metabolic pathways at the mRNA level because they are metabolic; therefore, they are independent of genomic controls.

Current methods for molecular metabolic modeling rely on enzyme kinetics data [29, 30] or on gene expression analysis [31]. Most of the research on peanut had focused on genetic and plant breeding logics of investigation. The approach adopted hereunder is different being based on molecular permutation modeling of metabolism at the mRNA level [20]. In the light of transcript silencing by homologous RNAs synthesized by GDH [32], it is predicted that induction of GDH isomerization and RNA synthesis by mineral ion treatments of peanut will promote arachin and fatty acid desaturase loss-of-functions leading to production of several metabolic variants that are substantially free of arachins and fatty acid desaturases. GDH is a target site of mineral ion action in plants [23]. Results presented hereunder show that treatment of peanut with phosphate plus potassium ions induced GDH to synthesize isomeric RNAs that coordinately silenced the mRNAs encoding Arachin h1, and fatty acid desaturase thereby producing Arachin-free low linoleic acid peanut. Molecular permutation of mRNAs could find expanded applications in the definition of differential gene expression [33] in metabolic disease conditions.

## Experimental Procedures

### Treatment of Peanuts with Mineral Ion Solutions

Peanut (*Arachis hypogaea* L. Cv. Virginia) seeds purchased from the grocery store were planted in 243.84 × 243.84 × 30.48 cm (width × length × depth) boxes, each filled with 18 bags of Metro-Mix 700 peat moss [25]. The applied mineral ion compositions were based on the model combinations in Table 1 that mimic the binomial subunit polypeptide compositions of the GDH isoenzymes [28]. The first box was left as the untreated control; the second box (N) was treated with 1 L of NH_4_Cl solution (25 mM); the third box (Pi) was treated with 1 L of Na_3_PO_4_ solution (20 mM); the fourth box (S) was treated with 1 L of Na_2_SO_4_ solution (50 mM); the fifth box (K) was treated with 1 L KCl solution (4 mM); the sixth box (NPKS) was treated with 1 L of combined NH_4_Cl (25 mM), Na_3_PO_4_ (20 mM), Na_2_SO_4_ (50 mM), and KCl (4 mM) solution; the seventh box (PK) was treated with 1 L of combined Na_3_PO_4_ (20 mM) and KCl (4 mM) solution; the eighth box (NS) was treated with 1 L of combined NH_4_Cl (25 mM) and Na_2_SO_4_ (50 mM) solution; the ninth box (PN) was treated with 1 L of combined Na_3_PO_4_ (20 mM) and NH_4_Cl (25 mM) solution; the tenth box (PS) was treated with 1 L of combined Na_3_PO_4_ (20 mM) and Na_2_SO_4_ (50 mM) solution. The boxes were watered every other day. Mineral nutrient solutions were applied sequentially, first at pre-flowering stage (2 weeks after seed germination), second at flowering, and third at post-flowering. When the leaves turned yellow (peanut maturity), pods were harvested, allowed to dry on the greenhouse floor for about 2 weeks; shelled by hand, and the kernels (seeds) weighed and stored at −30 °C.

**Table 1:** Some mineral salt combinations mimicking GDH subunit compositions

	Na_2_SO_4_ (50 mM)	KCl (4 mM)	Na_2_HPO_4_ (20 mM)	NH_4_Cl (25 mM)
N (25 mM)	NS	KN	PN	NN
P (20 mM)	PS	PK	PP	–
K (4 mM)	KS	KK	–	–
S (50 mM)	SS	–	–	–
NPK	NPKS	NPKK	NPPK	NNPK

### Protein Extraction and Electrophoresis

Peanut proteins were extracted from 10 g of control or mineral-treated seeds by homogenizing with 80 mL 10 mM Tris–HCl solution containing 0.002 % NaN_3_. Protein precipitated between 20 and 55 % ammonium sulfate saturation was pelleted by centrifugation (20,000×*g*, 20 min, 10 °C). The pellet was dissolved in minimum volume of extraction buffer, and dialyzed for 48 h at 8 °C against four changes of 10 mM Tris–HCl solution, each change being 4.5 L. Protein content was determined by Lowry method using bovine serum albumin for the calibration. Aliquots of extracts containing equal weights of protein (30–32 μg) were prepared and electrophoresed through sodium dodecyl sulfate (SDS) 12 % polyacrylamide gel using Bio-Rad Protean II cell until the bromophenol blue dye had migrated out of the gel. The electrophoresed gel was thereafter stained with silver solution. Arachin band intensities were digitalized using UN-SCAN-IT gel digitalizing software (Silk Scientific, Inc., Orem, UT, USA).

### Analyses for Fatty Acids

Dry and milled (composited) seeds (100 g) per experimental treatment, sent to SGS North America Inc., St. Rose, LA, USA were custom analyzed by HPLC for fatty acid composition.

### Purification and Assay of GDH

GDH charge isomers were purified by electrophoresis as described before [22] from peanut seeds harvested from the control or mineral salt-treated boxes. RNA synthetic activity of GDH isoenzymes [28] was assayed in combined deamination and amination substrate solutions of 0.1 M Tris–HCl buffer (pH 8.0) containing the four NTPs (0.6 mM each), CaCl_2_ (3.5 mM), l-glu (3.23 μM), NAD^+^ (0.375 μM), NH_4_Cl (0.875 mM), α-ketoglutarate (10.0 mM), NADH (0.225 mM), 5 units RNase inhibitor, 1 unit DNase 1, and 5 μg of actinomycin D. Reaction was started by adding 0.2 mL of whole gel-eluted GDH charge isomers containing 3–9 μg protein per milliliter. Final volume of the reaction was brought to 0.4 mL with 0.1 M Tris–HCl buffer pH 8.0. Reactions were incubated at 16 °C overnight and stopped by phenol-chloroform (pH 5.5) extraction of the enzyme. RNA was precipitated with ethanol, and dissolved in minimum volume of molecular biology quality water. GDH-synthesized RNA yield and quality [20] were determined by photometry and by agarose gel electrophoresis. Assays were carried out in duplicate to verify for reproducibility of the results.

### Total RNA

Total RNA was extracted from peanut seeds harvested from the control or mineral-treated boxes using the acidic phenol/chloroform (pH 4.5) method [34].

### Complementary DNA Synthesis, Cloning, Characterization, and Probe Selection

cDNAs were synthesized with 2 μg of each product RNA synthesized by the whole gel-eluted GDH charge isomers using random hexamer primer. Restriction fragment PCR amplification; adapter ligation; sequencing gel fractionation; and purification of cDNA fragments [28] were conducted according to the methods of Display Systems Biotech, Vista, CA, USA. Selected cDNA fragments were subcloned into pCR4-TOPO vector and transformed into TOP10 One Shot Chemically Competent *Escherichia coli* (Invitrogen, Carlsbad, CA, USA), followed by overnight growth on selective plates. Up to 10 positive transformant colonies were picked per plate and cultured overnight in LB medium containing 50 μg/mL of kanamycin. Plasmid DNA was purified with a plasmid kit (Novagen, Madison, WI, USA). The insert cDNA was sequenced with T3 and T7 primers by Genemed Synthesis, Inc. (South San Francisco, CA, USA), and Functional Biosciences, Inc. (Madison, WI, USA). To identify the GDH-synthesized RNAs that were homologous to mRNAs encoding fatty acid desaturases and arachins, the cDNA sequences were used as queries to search the NCBI nucleotide–nucleotide (excluding ESTs) BLAST (BLASTn), and non-redundant protein translation (BLASTx) databases. cDNAs that displayed the highest alignment scores with mRNAs encoding the fatty acid desaturases, and arachins were selected as the probes.

### Northern Blot Analysis

Equal amounts (10 μg) of total RNA, and RNAs synthesized by GDH charge isomers from the control and mineral salt-treated peanuts were loaded, briefly electrophoresed on 2 % agarose gels, stained with ethidium bromide, and photographed to verify RNA quality. RNA was electro-transferred from the electrophoresed gel onto Brightstar-Plus nylon membrane (Applied Biosystems, Foster City, CA, USA) as described before [27].

The cDNAs that were used as Northern probes were those homologous to mRNAs encoding arachins and fatty acid desaturases. For the labeling of the cDNA probes, cDNA inserts were amplified by PCR from the corresponding plasmids (15 ng) using M13 forward and M13 reverse primers (2 μM each), [^32^P]-dATP (6,000 Ci/mmol, 20 mCi/mL), dCTP/dGTP/TTP mix 50 mM, (2 μL), and Taq polymerase (1U), in a final volume of 50 μL. Amplification was according to Display Systems Biotech (Vista, CA, USA) “touch-down” PCR procedure (denature, 94 °C, 1 min; for the first 10 cycles, 94 °C, 30 s; anneal, 60 °C, 30 s; for the first cycle, then reduced the temperature 0.5 °C each cycle until an annealing temperature of 55 °C was reached after 10 cycles; extension, 72 °C, 1 min. Continued after another 25 cycles with 94 °C, 30 s; 55 °C, 30 s; 72 °C, 1 min; final extension, 72 °C, 5 min). Nylon membranes with immobilized RNA were prehybridized with ULTRAhyb buffer and hybridized with ^32^P-labeled cDNA inserts as probes overnight at 68 °C as described before [27]. Solutions of labeled cDNA were first heated in boiling water bath for 10 min before adding to the prehybridized membrane. After hybridization, the membranes were washed (30 min, 68 °C) with NorthernMax (Applied Biosystems, Foster City, CA, USA) low stringency wash solution followed by NorthernMax high stringency wash solution (30 min, 68 °C). The membrane was autoradiographed by exposure to X-ray film within intensifying screens at −80 °C. Northern band intensities were digitalized using UN-SCAN-IT gel digitalizing software (Silk Scientific, Inc., Orem, UT, USA).

## Results and Discussion

### Mineral Salt Treatments

Mineral ion compositions [28, 35] were formulated to mimic the stoichiometric ratios of, and to synchronize the subunit polypeptide compositions of GDH (Table 1). This made for internal repeats in the mineral ion compositions thus limiting stochastic variability in peanut plot treatments, imposed a firm control on the number of experimental repeats, and consolidated the biological comprehensiveness of the project design [27]. The peat moss was common to all the experimental boxes, thereby minimizing variability in soil physical and chemical characteristics. The control box without applied mineral ions was the negative control; whilst the NPKS-treated box was the positive control. Mineral treatments were applied after seed germination, at flowering, and after flowering so that at all times the mineral concentration in the soil was nearly constant and in that way, the effects of the mineral ions on GDH isomerization did not attenuate and/or fluctuate most of the time (synchronization). The triple application of mineral ions also functioned as reiterated triple dose replication to agree with the subunit ratios of GDH. These conditions assured the most efficient production metabolism of the peanuts as natural bioreactor through synchronization in the isomerization of GDH, synthesis of RNA by GDH, silencing of mRNAs by homologous RNAs synthesized by GDH, structural and spatial permutation of the metabolic pathways [20]. GDH isomerization [36] and synthesis of RNA as the target sites of mineral ion action are due to the binomial distribution of its three subunits in the hexameric isoenzymes, on the basis of the twin non-allelic GDH_1_ and GDH_2_ gene structure, with the gene (GDH_1_) encoding the more acidic subunits (α and a) being heterozygous and codominant, whereas the other gene (GDH_2_) encoding the less acidic subunit (β) is homozygous [37].

### Responses of Arachin Composition to Mineral Ions

**Fig. 1:**
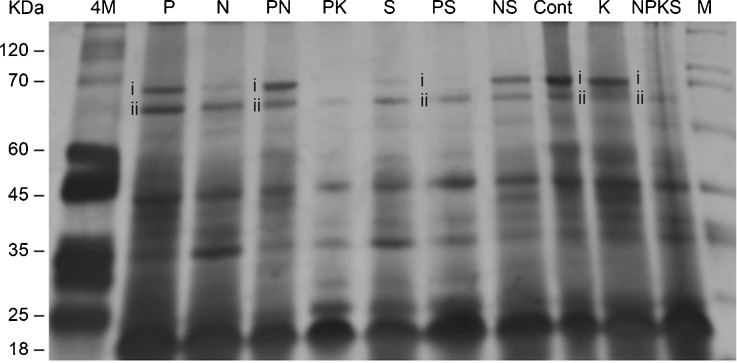
Sodium dodecyl sulfate–polyacrylamide gel electrophoresis of peanut proteins containing the arachin allergenic proteins. Aliquots containing equal (∼31 μg) weights of proteins extracted from the control and mineral salt treated peanuts were prepared and loaded into the wells of SDS-12 % polyacrylamide gel. The marker proteins were Sigma wide range protein molecular weight marker. After electrophoresis, the gel was silver stained. The arachin h1 bands are labeled *i* and *ii*

Virginia-type peanut arachin h1 proteins of ∼64–68 kDa [17] existed as two distinct isoforms (Fig. 1). Gel digitalization of the protein bands showed that the arachin isoforms constituted less than 1 % of the total protein of the peanut. Some peanut varieties may contain all allergens up to 5 % of the total protein [1]. The arachin h1 isoforms were differentially present in the peanuts, with the control, PN-, Pi-, NS-, and K-treated peanuts possessing the highest, while PK-, NPKS-, PS-treated, etc. had next to zero compositions (Fig. 1). Some of the arachin h2 and Ara h3 protein bands were differentially present (not annotated in Fig. 1) although not the focus of this presentation.

### Responses of Fatty Acid Composition to Mineral Ions

Palmitic, palmitoleic, stearic, arachidonic, linolenic, gadolenic, behenic, erucic, and lignoceric acid compositions were little affected by the mineral ion treatments of the peanut (Table 2). But oleic and linoleic acids changed epileptically according to the mineral ion regimen. The oleate and linoleate compositions changed from low-normal O/L (1.26–1.38) ratios in the NS-, PN-, Pi-, control, and NH_4_Cl-treated peanuts; through mid-normal (1.60–1.81) ratios in PS, S, K, and NPKS-treated peanuts; to high-normal O/L ratio of 3.31 in the PK-treated peanut. These trends of the O/L ratios suggested the occurrence of molecular reactions mimicking partial genetic changes of the wild genotype to the single functional homozygous mutations, and progressing precipitously towards the double mutation on both A and B genomes. Oleate and linoleate are metabolically directly linked so that expressing the O/L ratio reflects the relative contents of the two and possibly the genotype of the peanut cultivar [16, 38, 39].

**Table 2:** Virginia type peanut fatty acid composition and O/L ratios

Mineral ion treatments	PN	PK	S	P	Control	PS	NPKS	K	N	NS
Fatty acid composition % of total										
C16:0	10.0	8.3	8.6	9.6	9.7	8.7	8.6	8.7	9.5	9.8
Palmitic acid										
C16:1	0.1	0.1	0.1	0.1	0.1	0.1	0.1	0.1	0.1	0.1
Palmitoleic acid										
C18:0	2.2	3.3	2.5	2.1	2.2	2.2	2.2	2.7	2.2	2.1
Stearic acid										
C18:1	45.4	62.3	52.3	46.3	46.4	50.1	52.1	52.8	46.7	44.5
Oleic acid										
C18:2	35.0	18.8	28.9	34.5	34.4	31.4	29.6	29.2	33.9	25.2
Linoleic acid										
C18:3	0.1	<0.1	0.1	0.1	<0.1	0.1	0.1	<0.1	<0.1	0.1
Linolenic acid										
C20:0	1.2	1.5	1.3	1.2	1.3	1.2	1.1	1.3	1.3	1.2
Arachidic acid										
C20:1	1.7	1.7	1.7	1.7	1.7	1.8	1.9	1.6	1.8	1.9
Gadoleic acid										
C22:0	2.8	2.6	2.8	2.7	2.8	2.8	2.7	2.4	3.0	3.3
Behenic acid										
C22:1	0.1	0.1	0.1	0.1	0.1	0.1	0.1	0.1	0.1	0.1
Erucic acid										
C24:0	1.4	1.3	1.4	1.4	1.4	1.4	1.4	1.1	1.5	1.7
Lignoceric acid										
Oil, wt%	44.0	31.8	41.5	42.0	44.5	43.6	37.9	41.7	42.6	44.9
O/L ratios	1.29	3.31	1.80	1.34	1.35	1.59	1.76	1.80	1.38	1.26
% Unsaturated Fatty acids	82.4	82.4	83.2	82.8	82.7	83.6	84.0	83.8	82.6	82.0

### Structure of the Northern Probes

The GDH-synthesized RNA that shared plus/plus sequence homology with the mRNAs encoding the fatty acid desaturases was specifically homologous to the mRNAs encoded by FAD2A, FAD2B, and delta-12 fatty acid desaturase (Table 3) genes. This versatility enabled the cDNA of the RNA to monitor the differential abundance of those mRNAs. The cDNA of GDH-synthesized RNA that is homologous to the mRNA encoding arachin h1 allergen (Table 3) shares 77 % plus/plus sequence similarity with the probe that is homologous to the mRNAs encoding the fatty acid desaturases. The GDH synthesized RNAs that are homologous to the mRNAs encoding nitrate reductase, and NADH–glutamine oxoglutarate aminotransferase (GOGAT) [20, 28] share 73 and 79 % plus/plus sequence similarities, respectively, with the GDH-synthesized RNA that is homologous to the mRNAs encoding fatty acid desaturases (Table 3). These sequence similarities in the GDH-synthesized RNAs are their structural characteristics because the binomial subunit compositions of the enzyme and arrangement of subunits within the hexamers determine the frequency of the repeating, recurrent, inverted and non-recurrent sections of the synthesized isomeric RNAs [32].

**Table 3:** cDNAs of some GDH-synthesized RNAs homologous to mRNAs encoding arachin and fatty acid desaturase

(a) cDNA probe for mRNAs encoding Ara h I [*Arachis hypogaea*] isoforms: gb|AAB00861.1|; major allergen Ara h 1 [*Arachis hypogaea*]
GGGGCACCATACGCGCGATTCCCCTTAGGAGTCTGAACGAAGCGAACCCGATGTAGGAGGCGGGAAAACACGGAATGAGCGTCAACCAGGCGGTGAATGTTGGCCAGTTTTTGTATTGCCACCCGATGGAGATTATCTGCGGGGTGATGTTTCACCAGAACTTACTGCAGCGGGATTTCTGGACGAGACTTTTTCCACACGAACAGCCCCCAAAACAACCGCCCCCACAACTATAAGAAAGTTGAGGGAGGCAATGGTTCAAGACTCGTAAGGACGAAT
(b) cDNA probe for mRNAs encoding Fatty acid desaturase: gb|AF248739.1|AF248739 *Arachis hypogaea* delta-12 fatty acid desaturase gene;; HM 359250.1 FAD2A gene; HM 359252.1 FAD2B gene
GGAANCGGCGCNAATANGCANCTTGACGGTTCTNGTAATNCTGAGTTANCCGAATGNAGANNGCGGGCAAAACATGTGAANGTAGCGTNCAANCCAGGCCGTTAAAGGTTGCCGAGCTTTTGAAGTGCAACCCGATGGAGGTTATCTGCNGGGTGATGTTTCACCAGGACNTAATGGAGCGGGATTTCTGGACGGACATTTTCCAGCAGACNGTCACCGAAAACTACCGCCGCCACTACTTCAAGAAGGTTTAGGCAGGCTATCGGTCATGACTCANAAGGGCG

### Northern Blots

**Fig. 2:**
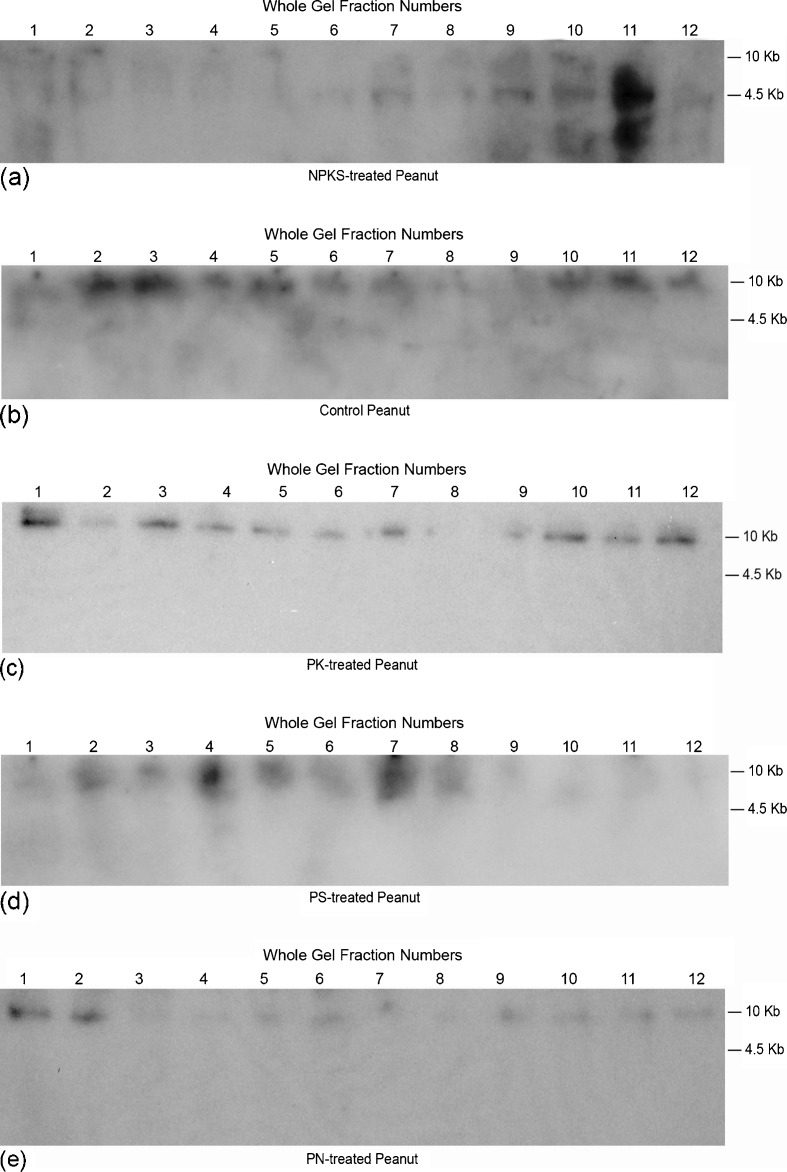
Northern analysis of GDH-synthesized RNAs that are homologous to the mRNAs encoding the arachin h1 allergens: equal weights (∼15 μg) of GDH-synthesized RNAs (*1*–*12*) by whole-gel purified GDH charge isomers of the N + P + K + S, control, PK-, PS-, and PN-treated peanuts were loaded into the wells of 2 % agarose gel and electrophoresed. Bio-Rad’s millennium RNA markers were loaded in the adjacent well. The electrophoresed gels were trans-blotted on to nylon membranes followed by membrane screening with ^32^P-labeled cDNAs of the GDH-synthesized RNA homologous to the mRNAs encoding the arachin h1. The membranes were washed with high stringency solutions and autoradiographed

**Fig. 3:**
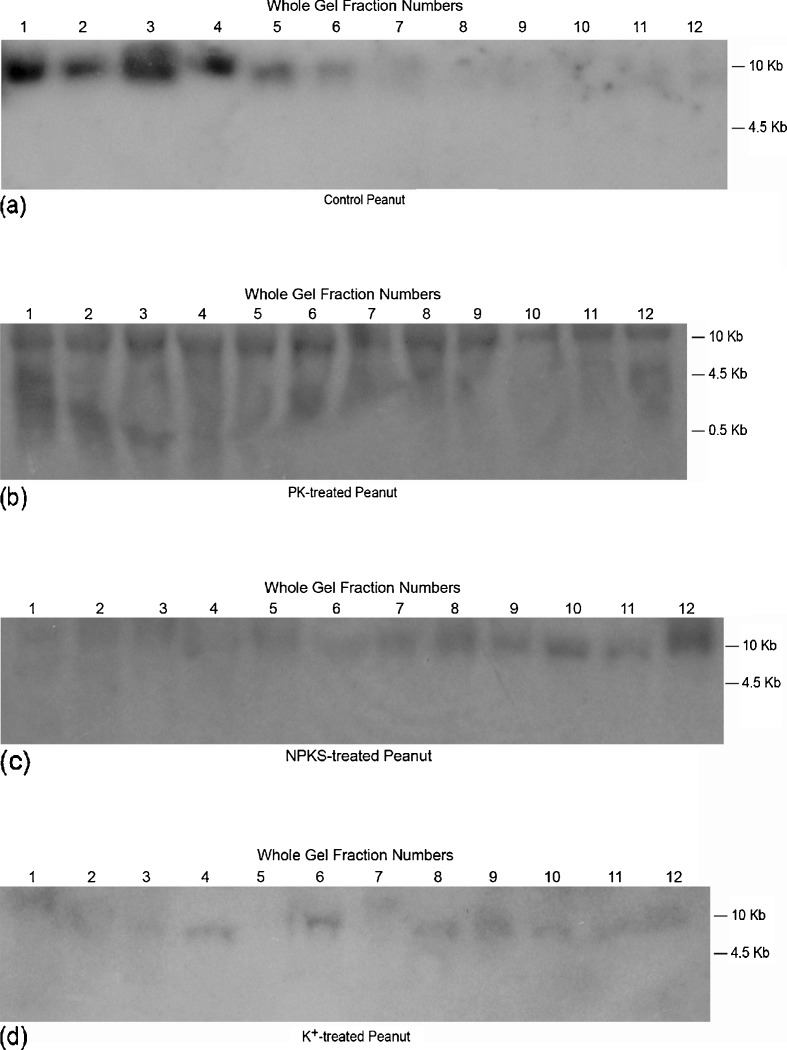
Northern analysis of GDH-synthesized RNAs that are homologous to the mRNAs encoding the fatty acid desaturases: equal weights (∼15 μg) of GDH-synthesized RNAs (*1*–*12*) by whole-gel purified GDH charge isomers of the control, PK, N + P + K + S, and K-treated peanuts were loaded into the wells of 2 % agarose gel and electrophoresed. Bio-Rad’s millennium RNA markers were loaded in the adjacent well. The electrophoresed gels were trans-blotted on to nylon membranes followed by membrane screening with ^32^P-labeled cDNAs of the GDH-synthesized RNAs homologous to the mRNAs encoding fatty acid desaturases. The membranes were washed with high stringency solutions and autoradiographed

Therefore, screening of the arrays of GDH-synthesized RNAs using the cDNAs of GDH-synthesized RNAs that were homologous to the mRNAs encoding arachin allergen, and fatty acid desaturases as probes produced Northern band patterns that displayed intensive similarities (Figs. 2 and 3). The Northern bands fell into the usual 0.5, 4.5, and >10 kb molecular weight groups [20]. The high molecular weight GDH-synthesized RNAs are suggested to undergo degradation to the low molecular weight oligos, which are the effective siRNA molecules [28]. The Northern bands demonstrated the population distribution of short sequences of GDH-synthesized RNAs (probes for the mRNAs encoding arachins and fatty acid desaturases) that were homologous to the GDH-synthesized RNAs; and the threshold quantities of probe sequences for silencing the mRNAs encoding arachin allergen, and fatty acid desaturases per mineral-treated and control peanuts. The arachin-related Northern band patterns (Fig. 2) for the GDH-synthesized RNA of SO_4_^−2^, and NH_4_Cl-treated peanuts (figures not shown) were similar to that of the PS-treated peanut. The GDHs of Pi-, K-, and NS-treated peanuts did not synthesize RNAs that were homologous to the mRNAs encoding the arachin allergens.

Fatty acid desaturase-related Northern band patterns (Fig. 3) for the GDH-synthesized RNA of SO_4_^−2^, and NS-treated peanuts (figures not shown) were similar to that of the NPKS-treated peanut. The GDHs of Pi-, PS-, PN-, and NH_4_Cl-treated peanuts did not synthesize RNAs that were homologous to the mRNAs encoding the fatty acid desaturases.

**Fig. 4:**
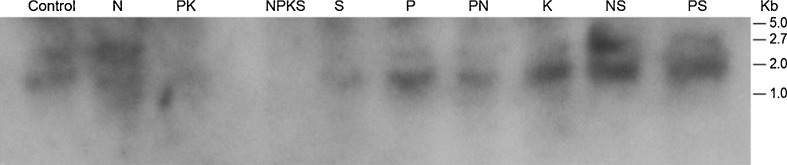
Northern analysis of total RNA for the mRNAs encoding arachin h1 allergen: equal (∼10 μg) weights of total RNA extracted from control and mineral-treated peanuts were loaded into the wells of 2 % agarose gel and electrophoresed. Bio-Rad’s millennium RNA markers were loaded in the adjacent well. The electrophoresed gel was trans-blotted on to nylon membranes followed by membrane screening with ^32^P-labeled cDNAs of the GDH-synthesized RNA homologous to the mRNAs encoding the arachin h1. The membranes were washed with high stringency solutions and autoradiographed. Note the dance (permutation) of the twin mRNAs

**Fig. 5:**
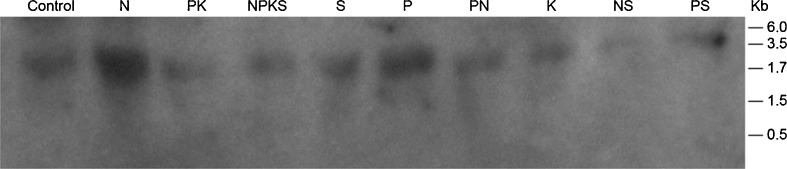
Northern analysis of total RNA for the mRNAs encoding fatty acid desaturases: equal (∼10 μg) weights of total RNA extracted from control and mineral-treated peanuts were loaded into the wells of 2 % agarose gel and electrophoresed. Bio-Rad’s millennium RNA markers were loaded in the adjacent well. The electrophoresed gel was trans-blotted on to nylon membranes followed by membrane screening with ^32^P-labeled cDNAs of the GDH-synthesized RNA homologous to the mRNAs encoding the fatty acid desaturases. The membranes were washed with high stringency solutions and autoradiographed. Note the dance (permutation) of the twin mRNAs

Screening of total RNAs using the cDNAs of GDH-synthesized RNAs that were homologous to the mRNAs encoding arachin h1, and fatty acid desaturases produced dissimilar Northern band patterns (Figs. 4 and 5) in contrast to the GDH-synthesized RNA Northern band patterns (Figs. 2 and 3). Two distinct mRNAs (∼1.8, and ∼2.2 kb) encoded the arachin h1 allergen isoforms (Fig. 4). Digitalized band results suggested that the two mRNAs were present in approximately equal proportions in those peanuts (control and NS-treated) with the highest compositions of arachin h1 allergen, but their proportions declined at different rates until nearly zero in those peanuts (PK and NPKS treated) with the lowest compositions of the allergen. A group of mRNAs (∼1.7 to ∼3.4 kb) encoded the fatty acid desaturases (Fig. 5). Digitalization suggested that the ∼1.8 kb bands of the fatty acid desaturase encoding mRNA populations of the control, SO_4_^−2^-, Pi-, PN-treated peanuts were preponderantly of the FAD2A and FAD2B genes. Similarly, the ∼3.4 kb band of the fatty acid desaturase encoding mRNA populations of the NS, and PS-treated peanuts were preponderantly of the delta-12 fatty acid desaturase (Fig. 5). Therefore, members of the group of mRNAs were present at differential ratios until their proportions were near to zero in the PK-treated peanut with high-normal O/L ratio (Table 2).

In both the arachins and fatty acid desaturases, the transitions from the equal proportions of their encoding mRNAs through differential reprogramming of the mRNAs until almost zero presence in some treated peanuts with concomitant attenuation of the arachin h1, and linoleate compositions are the phenomena that beg for explanation.

### Downregulation of the mRNAs Encoding the Arachins and Fatty Acid Desaturases

In the GDH-synthesized RNA Northern blots (Figs. 2 and 3), those peanuts (NH_4_Cl-, NS-, Pi-treated, etc.) that their GDHs did not synthesize RNAs homologous to the mRNAs encoding the arachins and fatty acid desaturases, the arachin levels were the highest (Fig. 1), and the O/L ratios were the lowest normal (Table 2), thus suggesting that the respective mRNA were not downregulated by GDH-synthesized RNA. Based on the extensive genotyping and fatty acid research by [14–16], the low-normal O/L ratios of the control, NH_4_Cl-, NS-, Pi-treated, etc. Virginia-type peanut used in this investigation suggested it was likely the OL_1_OL_1_OL_2_OL_2_ wild-type genotype. In addition, the near absence of the FAD2A and FAD2B encoded mRNAs accompanied by the low O/L ratios of the fatty acids of the NS- and PS-treated peanuts, suggested that the predominant presence of the mRNA encoding the delta-12 fatty acid desaturase produced active desaturase (Fig. 5). The role of delta-12 fatty acid desaturase encoded by the 3.5-kb mRNA has tended to be ignored in some published literature.

All the peanuts in which their mRNAs encoding the arachins (Fig. 4) and fatty acid desaturases (Fig. 5) displayed progressively declining disproportional levels suggested differential and partial mRNA downregulation. Messenger RNAs are downregulated by GDH-synthesized RNAs homologous to them [20], a function of GDH-synthesized RNA since evolutionary time [25]. Why were the mRNAs in each case not fully downregulated by the GDH-synthesized RNA? The answers reside in the cross-talk between the mRNAs encoding the arachins and fatty acid desaturases. The GDH-synthesized RNAs homologous to the arachin h1 and fatty acid desaturase encoding mRNAs shared 77 % plus/plus sequence similarity. The two sequences of GDH-synthesized RNAs (Table 3) could cross-recognize the other mRNAs albeit imperfectly thereby protecting them from knockout by the perfect homologous RNA. This knockdown rather than knockout of the mRNAs encoding the fatty acid desaturases tended to partially and metabolically convert the OL_1_OL_1_OL_2_OL_2_ genotype of the control Virginia-type towards mixtures of ol_1_ol_1_OL_2_OL_2_ and OL_1_OL_1_ol_2_ol_2_ metabolic variants (S-, K-, NPKS-treated peanuts) with mid-normal (1.60–1.81) O/L ratios (Table 2). That cross-talk may also account in part for the inability of the GDH-synthesized RNA homologous to the fatty acid desaturases to produce the ol_1_ol_1_ol_2_ol_2_ metabolic variant with an O/L ratio higher than that of the PK-treated peanut (Table 2). The mRNA encoding fatty acid desaturases were further protected from knockout by the 79 % plus/plus sequence similarity with the GDH-synthesized RNA homologous to the mRNA encoding NADH–GOGAT in the Pi- and PS-treated peanuts and 73 % plus/plus sequence similarity with GDH-synthesized RNA homologous to the mRNA encoding nitrate reductase in control, PK-, K-, S-, Pi-, and NS-treated peanuts [25]. The mRNAs encoding the arachin h1 isoforms were similarly protected from knockout by the 80 % plus/plus sequence similarity with the GDH-synthesized RNA homologous to the mRNA encoding NADH–GOGAT in the Pi- and PS-treated peanuts; and 73 % plus/plus sequence similarity with GDH-synthesized RNA homologous to the mRNA encoding nitrate reductase in control, PK, K, S, Pi, and NS-treated peanuts [25]. The cross-talks between arachin h1 biosynthesis, fatty acid desaturation, GS–GOGAT cycle, and nitrate reduction prevented the simultaneous shut down of all of nitrogen assimilation, arachin biosynthesis, and fatty acid desaturation under any prevailing mineral ion concentration and combination. This was part of the molecular metabolic adaptation that has allowed peanuts to produce oil in large quantities, different mineral ion conditions notwithstanding [25].

Although the GDH-synthesized RNAs homologous to the mRNAs encoding nitrate reductase, NADH–GOGAT, arachins, and fatty acid desaturases shared extensive plus/plus sequence similarities, their hybridization to GDH-synthesized RNA arrays [20, 25, 28] produced dissimilar banding patterns thereby emphasizing their individual identities. Digitally (UN-SCAN-IT) quantitated Northern bands (Figs. 1, 2, 3, and 4) showed that GDH synthesized the required RNA sequences in sufficient quantities to meet the thresholds for silencing the target homologous mRNAs. The minimum 2:1 ratio between the GDH-synthesized RNA and the target mRNA was the requisite threshold for normalizing [35] the silencing reaction. The ratio was the baseline control platform applied for unbiased judgment of the Northern bands across experimental repeats and mineral ion treatments. The preponderant >10 kb GDH-synthesized RNA in the Northern blots, although not translatable assured efficiency of transcript silencing by inducing maximum structural interaction with the homologous mRNAs, a crucial tertiary stabilization for the initiation of silencing [40]. Most of the hybridization reactions (Figs. 1, 2, 3, 4) gave more than a product per RNA lane. This was caused by partial incomplete downregulation of some mRNAs by homologous RNAs synthesized by GDH, and the isomeric nature of GDH-synthesized RNAs [32]. Therefore, high-throughput screening procedures (microarray, quantitative PCR, capillary electrophoresis, etc.) were inadequate for analyzing the hybridization reaction products.

### Molecular Modeling of Arachin and Fatty Acid Metabolism

**Fig. 6:**
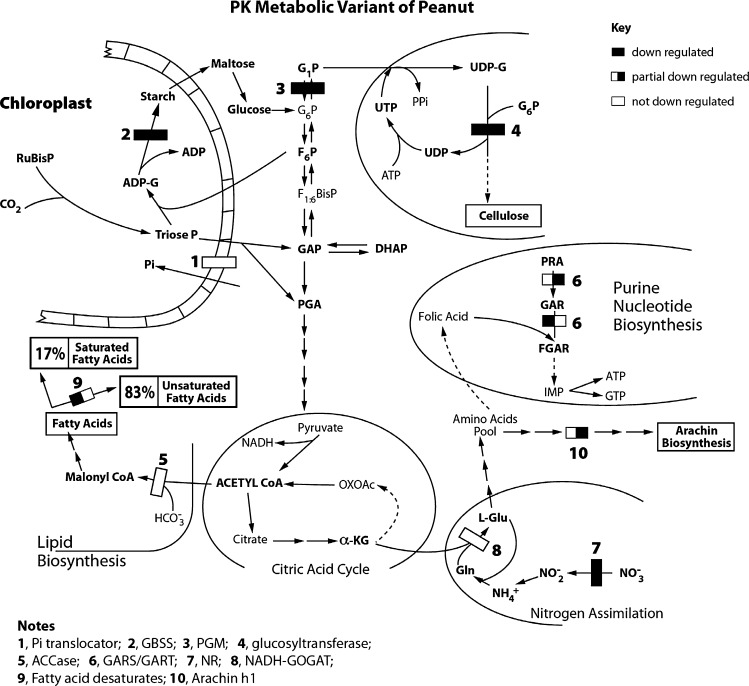
Molecular modeling of metabolism in PK-treated peanut to deplete the arachin and linoleic acid. Sketch of simplified metabolic pathways highlighting the enzymes whose encoding mRNAs were permuted by the RNAs synthesized by the GDH of the PK-treated peanut. Only the chloroplastic GBSS is shown. Only the cytosolic PGM is shown. Only the plastidial ACCase is also shown. Fatty acid desaturases are endoplasmic/microsomal. Arachin is endoplasmic. Because under the PK mineral ion concentrations, the mRNA {1} encoding Pi translocator was not downregulated, there was unlimited Pi translocator activity to import Pi into chloroplasts; mRNA {2} encoding GBSS was downregulated, phosphorylated C3, C5, or C6 compounds [42] were not expended in starch synthesis thereby priority channeling of triose phosphates to power fatty acid production. Also the mRNA encoding PGM {3} was downregulated thereby blocking utilization of glucose-6-phosphate for cellulose biosynthesis. Nitrogen assimilation is important in doubling peanut yield [30], therefore PK mineral ion composition ensured that the mRNA {8} encoding NADH–GOGAT was not simultaneously downregulated by the GDH-synthesized RNAs. This is aspect of the model sequential permutation of metabolism at the mRNA level by GDH-synthesized RNA to silence arachin and fatty acid desaturase encoding mRNAs without blocking fatty acid and carbohydrate metabolism under PK mineral ion conditions. *G*_*1*_ *P* glucose-1-phosphate, *G*_*6*_ *P* glucose-6-phosphate, *α-KG* alpha-ketoglutarate

The signaling at the mRNA level between arachin biosynthesis and fatty acid desaturation on one hand, and with nitrogen assimilatory and primary metabolic pathways [20, 25] on the other hand introduce the concept of probability or likelihood for metabolites to flow into oleic acid, linoleic acid, arachin isoforms, or to alternative directions in peanuts treated with mineral ions. The cross-talk between arachin biosynthetic and fatty acid desaturation pathways are biochemically logical because they depend on the rational partitioning and differential metering of the products of nitrogen and CO_2_ assimilation, saccharide biosynthesis and glycolysis for their operation [30]. Metabolic pathway integration at the mRNA level was consolidated by statistical redistribution of metabolic intermediates to molecularly connected pathways. GDH-synthesized RNAs are statistical in nature, their primary structure being dependent on the binomial subunit arrangement of the GDH isoenzymes [27]. Therefore, several probabilities arise under a prevailing natural environment (mineral nutrient combination/concentration) for the GDH-synthesized RNA to reprogram the mRNA abundances and consequently the accumulation of saturated and unsaturated fatty acids, arachins, and other yield components in the biological system. The reprogramming of the sequence of metabolism in response to the prevailing mineral ions in order to maximize accumulated yield can best be approached by permuting the yield-specific pathways guided by the mRNA systems that were not downregulated, partially downregulated, and fully downregulated by GDH-synthesized RNAs per mineral environment and limited to the 10 metabolic pathways affected (Fig. 6).

Digital quantitation of Northern bands (Fig. 4) suggested that the mRNAs encoding the arachin h1 isoforms in control, NS-, Pi-, K-, and PN-treated peanuts were not downregulated; in NPKS-, S-, PS-, NH_4_Cl-, and PK-treated peanuts were partially downregulated. Similarly, the mRNAs encoding the fatty acid desaturases (Fig. 5) in control, NS-, Pi-, PN-, and NH_4_Cl-treated peanuts were not downregulated; in NPKS-, S-, PS-, K-, and PK-treated peanuts were partially downregulated. None of the mineral salt conditions induced the full downregulation of the mRNAs encoding the arachins or the fatty acid desaturases.

The GDH-synthesized RNA probe (Table 3) was homologous to all the mRNAs encoding the arachin h1 isoforms (Fig. 4). This suggested that the arachin h1 encoding mRNAs were permuted among themselves. Similarly, the fatty acid desaturase encoding mRNAs (Fig. 5) were permuted among themselves. These sequential permutations differentiated the arachin (Fig. 1), oleic acid, and linoleic acid, etc. (Table 2) percentage compositions of one treated peanut from the other. Statistical dovetailing of arachin and fatty acid desaturation sequential permutations into the primary metabolic pathways (Fig. 6) led to spatial permutation by GDH-synthesized RNAs. The number of permutations of the 10 pathways varied widely: control peanut induced 210; NPKS-treated peanut induced 3,150; NS-, PS-, K-, and PN-treated peanuts each induced 1,260; Pi-, S-, and NH_4_Cl-treated peanuts each induced 2,520; PK-treated peanut induced 4,200 permutations. Therefore, there were many probable rearrangements of peanut metabolism in response to any mineral ion regimen. This was responsible for the inability of mineral salts to totally eliminate (knock out) the arachin compositions, or to knock out all the fatty acid desaturases. This is also the explanation for the conflicts about the peanut oleic acid responses to applied mineral nutrients [8, 11, 12, 16, 18]. Frequency distribution plot of the metabolic pathways versus the number of permutations gave a positively skewed graph reminiscent of the GDH binomial isoenzyme population distribution pattern [22]. This confirmed that the mineral ions (Table 1) targeted the peanut GDH. Only NPKS- and PK-treated peanuts fell to the right of the mean (2,520) in agreement with their near zero arachin compositions (Fig. 1) and below normal linoleic acid contents (Table 2). Thus, these results (Fig. 6) as different from computational approaches to metabolic profiling [29, 31] have quantitatively unified molecular biology and metabolic pathway kinetics into one simple biotechnology for improving the quality of peanut. In all the 10 environmental conditions studied, there was complete match between the mRNAs permuted and the compositions of arachins and fatty acids. Therefore, the metabolic pathways are intertwined at the mRNA level as they are at the enzyme/substrates/products level. This uncovers a new biological level in the hierarchically ordered systems of the biological world. Variations in the literature percentages of peanut fatty acids are due to reprogramming of the mRNAs encoding the fatty acid desaturases by GDH-synthesized RNA [14].

The consortium of downregulated, not downregulated, and partially downregulated reaction steps in the metabolic pathways acted in concert to normalize fatty acid metabolism so that the values of unsaturated (∼83 %) and saturated (∼17 %) fatty acids were similar in the mineral-treated peanut as in the control peanut (Table 2). The compensation between saturated and unsaturated fatty acid values is one of the evolutionary adaptations (Fig. 6) that enable peanut metabolism to produce large quantities of oil the adversity of the mineral ion environment notwithstanding [25].

## Discussion

The regulatory enzyme steps (Fig. 6) of the primary metabolic pathways [25] into which the arachin and oleic acid biosynthetic pathways were dovetailed included phosphate translocator, granule-bound starch synthase (GBSS), phosphoglucomutase (PGM), glucosyltransferase, acetyl CoA carboxylase (ACCase), glycinamide ribonucleotide (GAR) synthase/GAR transformylase, nitrate reductase, and NADH-glutamate synthase (GOGAT). Carbon dioxide assimilation is regulated in peanut [20] by Pi translocator, a chloroplast membrane protein antiport system that uses Pi and phosphorylated C3, C5, or C6 compounds as counter substrates [41]. Starch synthases are another point for regulation of CO_2_ assimilation in peanut. They catalyze the formation of α-(1–4)-linked linear glucosyl chains [42]. PGM is a regulatory step in peanut glycolysis. It partitions carbon between the pathways of starch synthesis and glycolysis [43]. Glucosyltransferase regulates peanut saccharide metabolism [28]. Cellulose biosynthesis involves chain initiation, elongation, and termination, with the participation of glucosyltransferase in the chain initiation reaction [44]. ACCase catalyzes the ATP-dependent carboxylation of acetyl-CoA to form malonyl-CoA, the precursor for fatty acid synthesis [45]. ACCase controls the production of fatty acids in peanut [25]. The free fatty acids produced are first esterified in preparation for soluble plastidial desaturation [46]. Purine biosynthesis, nitrate reduction, and GS-GOGAT cycle cooperate to assimilate nitrogen in peanut [20]. The salvaged NH_4_^+^ ion is utilized in metabolism including amino acid and protein (arachins etc.) biosyntheses. Therefore, Fig. 6 is a summary sketch of the regulatory steps in peanut primary metabolism.

The metabolic model for the PK-treated peanut (Fig. 6) shared several similarities at the level of mRNA regulation with that of the NPKS-treated peanut [25] with the exception of the mRNA encoding Pi transporter. The similar loss-of-function responses of arachin and fatty acid desaturases in the PK- and NPKS-treated peanuts (Fig. 1 and Table 2) are in accord with the 90 % similarities in the metabolic models of the two metabolic variants; whereas the not downregulated mRNA encoding Pi transporter in the PK-treated peanut accounted for the huge yield of fatty acid in contrast to the very low fatty acid yield in the NPKS-treated peanut [20]. These emphasize the crucial importance of Pi transporter in peanut CO_2_ assimilation. Therefore, PK and NPKS are the recommend mineral salt combinations for producing arachin-depleted and low linoleic acid peanut. Further advantage of this biotechnology is that it is developing country and small farmer friendly, without the need to purchase proprietary seeds and import large quantities of inorganic fertilizers.

As a new gateway into the biological world, procedures for analyzing mRNA downregulation by GDH-synthesized RNAs [20] are slightly different from classical siRNA approaches in that there are no transfection and reverse transcription steps, and the GDH-synthesized RNAs are endogenously elicited. Messenger RNA downregulation by GDH-synthesized RNA takes place as a biochemical reaction at the molecular level; and it is readily inducible by environmental conditions. Experimentation on GHD-synthesized RNA embodies many layers of controls, technical repeats, normalization, and standardization. GDH purification by sub-zero temperature whole gel electro-elution into the 14 chambers of Bio-Rad’s mini whole gel eluter gave 14 purified instead of one preparation of the enzyme. The binomial assembly of the subunit polypeptides in the hexameric isoenzymes did not permit the combination of the 14 purified fractions to make a single preparation. Therefore, in these protocols, there were 14 repeats of the purified GDH, 14 repeats of RNA synthesis reaction, and 14 repeats of RNA loading into agarose gel. The extensive repetitive steps in the GDH experimentation demonstrate reproducibility of the synthetic reaction and are vividly portrayed by the similarities in the Northern blot lanes (Figs. 2 and 3). More than 90 % of the RNA lanes showed that RNA repetitive loadings onto gels were consistent, efficient and reproducible across mineral-treated peanuts [20, 25]. Such results evoke confidence in the research approach. The similarities of the GDH-synthesized RNA patterns on agarose gels and Northern blots notwithstanding, when the GDH-synthesized RNAs are converted to cDNAs followed by restriction enzyme digestion, the resulting fragments are homologous to hundreds of mRNAs [22]. Therefore, GDH-synthesized RNAs are different in the arrangement of the repeating, inverted, and recurrent isomeric sequences [27]. Northern blots were performed in duplicate using each cDNA of GDH-synthesized RNA as probe, thereby making a total of 28-repeat RNA tracks per probe. There were several lines of evidence for internal control reactions in the downregulation of mRNA, including the plus/plus and/or plus/minus sequence similarities among the GDH-synthesized RNAs [20], partial silencing of mRNAs encoding distinct enzymes etc. Messenger RNAs encoding several housekeeping enzymes were not useful references in the Northern assays because Northern results [27] in which GDH-synthesized RNAs were used as probes suggested the housekeeping mRNAs were also reprogrammed under the experimental regimen. Therefore, the ratio of 2:1 between the GDH-synthesized RNA and the target mRNA was adopted as the minimum normalization and standardization factor for silencing. Although the ideal reference internal mRNA should be one that does not vary as a function of experimental treatment, it has been difficult to identify a single mRNA/rRNA that meets the criterion [47]. In the total RNA Northern results (Figs. 4 and 5), where the mRNAs were partially silenced, it was assumed that the residual mRNA constituted ∼50 % of the total mRNA. The full amount of mRNA was determined when the GDH of the peanut did not synthesize the homologous RNA. Therefore, the total RNA Northern results pictorially represented the permutation of mRNAs by homologous GDH-synthesized RNAs. The limits of the biological comprehensiveness of the GDH-synthesized RNA experimentation were defined by the peanut without any mineral ion treatment representing the negative control and the peanut with the full mineral ion concentration and composition (NPKS) representing the positive control. Positive control is important to provide the line of verification for the molecular mechanism of GDH action and for result verification, confirmation, and validation. Although all the experimental peanuts were green, the control, NPKS, sulfate, potassium, N + S, and P + N-treated peanuts exhibited low growth vigor. The said comparative reluctance to grow was clearly manifested by the low qualities of the corresponding Northern blots for their GDH-synthesized RNAs (Figs. 2 and 3). Therefore, the quality of the GDH-synthesized RNA Northern results is somehow related to the vegetative growth vigor of the peanut. Figures 2 and 3 were among the first Northern blots not based on genomic RNA [20, 25, 28].

The cDNA probe (Table 3) for the mRNAs encoding the Ara h1 isoforms is unique in not sharing homology with those mRNAs encoding the Ara h2 and Ara h3 proteins. This helped to avoid possible complex molecular protein and RNA differential analyses that could have arisen had the arachin h1 cDNA probe been less specific. Permutation of the concentrations of all the mRNAs encoding the three arachins (Ara h1, Ara h2, Ara h3) would result to a comprehensive biotechnology for minimizing the arachin and the linoleate contents. Such a biotechnology would encourage small-scale farmers especially in the developing countries to produce healthy peanuts free from allergenic arachins and with low linoleic acid contents. A structural search of the GDH-synthesized RNAs for sequences that are specifically homologous to the mRNAs encoding the Ara h2, Ara h3, and other possible allergens is ongoing and is yet to identify them. GDH-synthesized RNA probes would be ideal for this biotechnology because they are metabolic [26] and isomeric with several internally repetitive and inverted sequences [32] that maximize their siRNA specificity for their target mRNAs.
